# IL-31 and IL-8 in Cutaneous T-Cell Lymphoma: Looking for Their Role in Itch

**DOI:** 10.1155/2021/5582581

**Published:** 2021-07-20

**Authors:** Maria Abreu, Marta Miranda, Mafalda Castro, Iolanda Fernandes, Renata Cabral, Ana Helena Santos, Sónia Fonseca, João Rodrigues, Magdalena Leander, Catarina Lau, Inês Freitas, Susana Coimbra, Alice Santos-Silva, Margarida Lima

**Affiliations:** ^1^Centro Hospitalar Universitário do Porto (CHUP), Porto, Portugal; ^2^Faculdade de Farmácia, Universidade do Porto (FFUP), Porto, Portugal; ^3^Serviço de Hematologia Clínica, Centro Hospitalar Universitário do Porto (CHUP), Porto, Portugal; ^4^Serviço de Dermatologia, Centro Hospitalar Universitário do Porto (CHUP), Porto, Portugal; ^5^Consulta Multidisciplinar de Linfomas Cutâneos e Mastocitoses (CMLC), Centro Hospitalar Universitário do Porto (CHUP), Porto, Portugal; ^6^Unidade Multidisciplinar de Investigação Biomédica, Instituto de Ciências Biomédicas Abel Salazar, Universidade do Porto (UMIB/ICBAS/UP), Porto, Portugal; ^7^Serviço de Hematologia Laboratorial, Centro Hospitalar Universitário do Porto (CHUP), Porto, Portugal; ^8^Cooperativa de Ensino Superior Politécnico e Universitário (CESPU), Instituto de Investigação e Formação Avançada em Ciências e Tecnologias da Saúde (IINFACTS), Gandra, Paredes, Portugal; ^9^Unidade de Ciências Biomoleculares Aplicadas (UCIBIO), Rede de Química e Tecnologia (REQUIMTE), Porto, Portugal

## Abstract

The itch associated with cutaneous T-cell lymphoma (CTCL), including Mycosis Fungoides (MF) and Sézary syndrome (SS), is often severe and poorly responsive to treatment with antihistamines. Recent studies have highlighted the possible role of interleukins in nonhistaminergic itch. We investigated the role of IL-31 and IL-8 in CTCL, concerning disease severity and associated itch. Serum samples of 27 patients with CTCL (17 MF and 10 SS) and 29 controls (blood donors) were analyzed for interleukin- (IL-) 31 and IL-8; correlations with disease and itch severity were evaluated. IL-31 serum levels were higher in CTCL patients than in controls and higher in SS than in MF. Also, serum IL-31 levels were higher in patients with advanced disease compared to those with early disease, and they correlated positively with lactate dehydrogenase and beta 2-microglobulin levels, as well as with the Sézary cell count. Itch affected 67% of CTCL patients (MF: 47%; SS: 100%). Serum IL-31 levels were higher in itching patients than in controls and in patients without itching. There was no association between serum IL-8 and disease severity, nor with itching. Serum IL-8 levels correlated positively with peripheral blood leukocyte and neutrophil counts in CTCL patients. Our study suggests a role for IL-31 in CTCL-associated itch, especially in advanced disease and SS, offering a rational target for new therapeutic approaches. Increased serum IL-8 observed in some patients may be related to concomitant infections, and its role in exacerbating itch by recruiting neutrophils and promoting the release of neutrophil proteases deserves further investigation.

## 1. Introduction

Cutaneous T-cell lymphomas (CTCLs), classically represented by Mycosis Fungoides (MF) and Sézary Syndrome (SS), account for 70% of cutaneous lymphomas and 10% of extranodal non-Hodgkin´s lymphomas [1]. These chronic pathologies reduce the patient´s quality of life [2], and the prognosis depends on the CTCL type and stage [3].

Pruritus, or itch, a frequent feature in CTCL, is particularly severe in patients with SS [4, 5], being often recalcitrant and refractory to treatment [6, 7], thus motivating the study of the pathways involved [8].

Histamine has been shown to cause itching [9], and antihistamines are the most prescribed medications for itch [10]. Nevertheless, CTCL-associated itching is usually refractory to antihistamines, suggesting alternative pathways to its genesis.

Molecules suspected to be involved in nonhistaminergic itch include neurotransmitters [11–16], lipid mediators [17–20], and proteases, via proteinase-activated receptors (PARs) [21], as well as cytokines and chemokines [22].

Among the cytokines potentially implicated is interleukin- (IL-) 31, produced mainly by T helper 2 cells (Th2) [23]. IL-31 signals through a heterodimeric receptor [24] and stimulates the JAK-STAT, RAS/ERK, and PI3K/AKT pathways [25]. It does not induce itch immediately after skin challenge [26] but has receptors in the dorsal root ganglia [27] and is a likely mediator in nonhistaminergic itch [28, 29].

Previous studies have suggested the involvement of IL-31 in itching diseases including atopic dermatitis (AD) [30–39], other pathological conditions [40–45], drug-induced itch [46], and neoplastic diseases, such as CTCL [47–53].

Some studies have reported increased IL-31 serum levels (sIL-31) in itching diseases, and most of them have provided evidence for a positive correlation between sIL-31 and itch severity in AD [31, 32, 34, 36] and CTCL [47, 49, 50], with some contradictory results [51, 52]. In addition, the expression of IL-31 mRNA, IL-31, and its receptors has been shown to be increased in AD [32, 33, 48] and CTCL [49, 53] skin lesions and to correlate with itch severity and disease stage. IL-31 receptors have also been described in the nerve fibers of the AD dermis and in normal dorsal root ganglia, which mediate the sensation of itch [38]. Finally, specific IL-31 gene polymorphisms have been associated with itch and AD severity [37, 39].

Bacterial infections, namely, skin infections, occur frequently in patients with CTCL, and bacteremia and pneumonia are frequent causes of death [54]; cutaneous colonization with staphylococci is common, especially in SS, worsening both erythroderma and itching [55, 56].

Microorganisms can induce and/or exacerbate the inflammatory responses due to secretion of proinflammatory cytokines, namely, IL-8 [57], involved in neutrophil chemotaxis [58, 59]. Increased IL-8 serum levels (sIL-8) have been described in AD [60] and psoriasis [61, 62], being higher in more severe conditions and improving with treatment. Increased IL-8 mRNA was also found in peripheral blood (PB) mononuclear cells of AD patients [63]. However, IL-8 does not induce itch upon cutaneous injection, and a direct role of IL-8 in itch is unlikely [64, 65]. Another study showed that sIL-8 did not correlate with pruritus in primary myelofibrosis [66].

Considering the prevalence and severity of itch in CTCL and its resistance to treatment, we decided to investigate the role of IL-31 and IL-8 in the pathophysiology of itch in CTCL.

## 2. Materials and Methods

### 2.1. Study Population

The study group included CTCL patients and healthy controls (blood donors). Patients with evidence of active infections or other concomitant neoplasms were excluded.

### 2.2. Clinical Data

Clinical data were obtained from hospital records and comprise lymphoma classification and staging, type and extension of the cutaneous lesions, the presence and intensity of itch, treatments, concomitant diseases, and past clinical history.

The diagnosis and classification of CTCL followed the recommendations of the European Organization for Research and Treatment of Cancer (EORTC)/World Health Organization (WHO) [67–69].

Lymphoma TNMB (tumor-node-metastasis-blood) staging was established using the ISCL/EORTC proposal [70].

Erythroderma was evaluated using a scale with 5 levels (0 = normal; 1 = barely detectable erythema and scaling; 2 = readily detectable erythema, edema, and scaling; 3 = marked erythema and skin exfoliation; and 4 = fissuring, maximal erythema, induration, and tumors) and quantifying the percentage of cutaneous area afflicted [71].

Itch severity was assessed through a visual analogue scale (VAS), ranging from the absence of itch (0 points) to the highest itch intensity (10 points). Arbitrarily, itch scaled from 1 to 5 points was considered mild/moderate and itch ranging from 6 to 10 points was considered intense/severe.

### 2.3. Laboratory Studies

Blood samples were collected into anticoagulant-free tubes for quantifying serum cytokines and into tubes containing ethylenediaminetetraacetic acid, for cell counts and lymphocyte immunophenotyping.

Serum cytokine levels were quantified through the LEGEND MAX™ Human IL-8 and IL-31 Enzyme-Linked Immunosorbent Assays (Biolegend, U.S.A.).

Blood cell counts were obtained through an automatic hematological counter (LH780, Beckman Coulter, U.S.A.). Confirmation of differential leukocyte count and the search for SC by morphology were performed through optic microscopy in PB smears (Leishman's stain).

Biochemical analysis included glucose, liver, and kidney tests, beta 2 microglobulin (B2MG), and lactate dehydrogenase (LDH), among others.

Lymphocyte immunophenotyping was made by flow cytometry, using 4- or 8-color staining with fluorochrome-conjugated monoclonal antibodies specific, at least, for CD2, CD3, TCR, CD4, CD5, CD7, CD8, CD26, and CD28 and completed with the study of TCR variable region beta chain repertoire (Immunotech, Beckman Coulter), as previously described [72]. Samples were read in a FACSCanto II flow cytometer (Becton Dickinson, U.S.A.) and analyzed though the Infinicyt program (Cytognos, Spain).

TCRG gene rearrangement studies were performed as previously described [73], using the TCRG Gene Clonality Assay (InVivo Scribe Technologies, U.S.A.) and following the Biomed II protocol [74]. Data were analyzed using the Peak Scanner Software v1.0 (Thermo Fisher Scientific, U.S.A.).

### 2.4. Statistical Analysis

Results were presented as relative and absolute frequencies for qualitative variables and as median, range, and mean ± standard deviation for continuous quantitative variables. Data distribution was evaluated by Kolmogorov–Smirnov analysis. For comparison between groups, we used, for continuous variables, the Mann–Whitney *U* test; for categorical variables, the chi-squared test was employed. Strength of correlations between variables was determined through Kendall's tau-B correlation coefficient. *p* values < 0.05 were considered statistically significant. Data analysis was performed with the Statistical Package for the Social Sciences (SPSS^®^), v23.

## 3. Results

Twenty-seven CTCL patients (median age: 66 years; 51.9% males) and 29 controls (median age: 58 years; 48.3% males) were included. Among patients, all having active disease, 17 (63%) were diagnosed with MF and 10 (37%) had SS (Table 1).

All patients had skin lesions of any sort, most often patches (63.0%), plaques (29.6%), and erythroderma (9 patients, all with SS). The affected skin surface area was <10% in 8 patients (29.6%) and ≥80% in 11 patients (40.7%), 9 of whom having SS. At the time of study, only 2 patients had palpable lymph nodes. In addition, 11 patients (40.7%) had SC in the PB; the percentage of SC among total lymphocytes was >5% in 10 cases (37.0%), and the SC count exceeded 1000 cells/*μ*l in 5 cases (18.5%), given blood involvement to be classified as B0b in 1 case, B1b in 5 cases, and B2b in another 5 cases. Using the TMNB staging, 9 patients were classified as stage Ia, 8 as stage Ib, 1 as stage IIb, 4 as stage IIIb, and 5 as stage IVa.

Eighteen patients (66.7%) complained of itch at the time of the study, this symptom being observed in all SS patients, and only in 47.8% of patients with MF. The median itch VAS score for all patients was of “1,” being of “1” for patients with early disease and of “10” for patients with advance disease (*p* < 0.001). The highest itch scores were found in SS patients, who had a median VAS score of “9,” as compared to “0” in MF (*p* = 0.001). Itch was classified as mild/moderate in 7 patients (25.9%) and as intense/severe in 11 patients (40.7%), with a higher proportion of SS patients referring intense/severe itch (80%), as compared to MF (17.6%).

Eighteen patients (66.7%) were under therapy directed at the disease: topical corticosteroids (10/27, 37.0%), methotrexate (4/27, 14.8%), oral bexarotene (2/27, 7.4%), oral corticosteroids (2/27, 7.4%), and extracorporeal photopheresis (1/27, 3.7%). As for symptomatic treatment and besides corticosteroids, 11 patients (40.7%) were taking antihistamines, 1 patient was medicated with mirtazapine, and another with aprepitant.

Eleven patients (40.7%) had concomitant pathologies, of whom 2 patients had psoriasis and 2 had alcoholic liver disease. In addition, 2 patients had a history of other neoplasm (carcinoma of the thyroid gland and gastric adenocarcinoma), in remission at the time of study.

The hematologic study (Table 2) showed 3 patients with leukocytosis and one with neutrophilia; 9 patients had lymphopenia, and 3 had lymphocytosis. Anemia was found in 7 patients, but only one had Hg ˂ 10 g/dL, who also presented thrombocytopenia.

Eleven patients (40.7%) (10 SS, 1 MF) had CD4+ lymphoma cells in the PB (Table 2). In these cases, the phenotypically abnormal CD4+ T cells represented a median value of 82.9% of the CD4+ T cells, 69.3% of T cells, 48.2% of lymphocytes, and 11.1% of WBC, being >5% of PB lymphocytes in 10 patients (27.0%), classified as B1 or B2. The median count of CD4+ lymphoma cells was of 604 × 10^6^/L, being ≥1000/*μ*l in 5 cases (B2) (18.5%), corresponding to patients with SS.

The malignant T cells were typically CD3+, TCR-alpha/beta+, CD4+, and CD5+, failed to express CD26, and frequently had abnormally low levels of CD3 and/or CD4, as well as low or absent CD7 expression (data not shown). The TCR-Vbeta family expressed by circulating SC was identified in 8/11 cases (72.7%), corresponding to TCR-Vbeta17.1 (*n* = 3), TCR-Vbeta5.1 (*n* = 2), and TCR-Vbeta3.1, TCR-Vbeta20.1, or TCR-Vbeta22.1 (1 case each).

Fifty percent of patients with CTCL had increased LDH (25.0% of MF cases and 80.0% of SS cases), and 34.8% had increased B2MG (21.4% of MF and 55.6% of SS cases) (Table 2). LDH and B2MG were significantly higher in SS, as compared to MF (*p* = 0.002 and *p* = 0.027, respectively). Abnormal liver tests were found in 2 patients with alcoholic liver disease; one patient had mild renal insufficiency.

### 3.1. Interleukin-31

**Figure 1:**
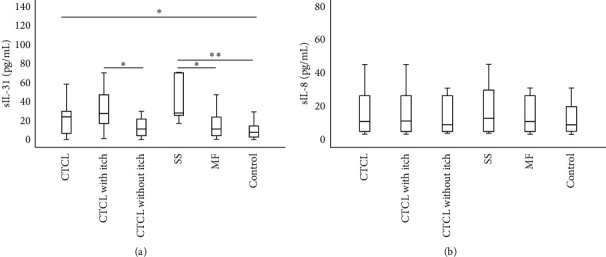
IL-31 (a) and IL-8 (b) serum levels according to diagnosis and to the presence of itch. In this figure, 2 outliers were excluded for sIL-31 (sIL-31 > 200 pg/mL) and 1 outlier was excluded for IL-8 (sIL-8 > 200 pg/mL). Statistical analysis was performed using the Mann–Whitney *U* test. ^*∗*^*p* < 0.05; ^*∗∗*^*p* < 0.01; and ^*∗∗∗*^*p* < 0.001. Statistics for sIL-31 (including/excluding the outliers): CTCL *vs*. controls: *p* = 0.012/*p* = 0.032; MF *vs*. controls: *p* = 0.333/*p* = 0.333; SS *vs.* controls: *p* < 0.001/*p* = 0.001; MF *vs*. SS: *p* = 0.004/*p* = 0.024; CTCL without itch *vs*. controls: *p* = 0.430/*p* = 0.649; CTCL with itch *vs*. controls: *p* = 0.002/*p* = 0.007; and CTCL with itch *vs*. CTCL without itch: *p* = 0.021/*p* = 0.048. Statistics for sIL-8 (including/excluding 1 CTCL outlier, corresponding to a patient with hyperleukocytosis and sIL-8 >200 pg/mL): CTCL *vs*. controls: *p* = 0.863/*p* = 0.946; MF *vs*. controls: *p* = 1.000/*p* = 1.000; SS *vs*. controls: *p* = 0.740/*p* = 0.893; MF *vs*. SS: *p* = 0.639/*p* = 0.958; CTCL without itch *vs*. controls: *p* = 0.783/*p* = 0.914; CTCL with itch *vs*. controls: *p* = 0.726/*p* = 0.973; and CTCL with itch *vs*. CTCL without itch: *p* = 0.743/*p* = 0.916. CTCL, cutaneous T-cell lymphoma; MF, Mycosis Fungoides; SS, Sézary syndrome; sIL-8, interleukin-8 serum levels; and sIL-31, interleukin-31 serum levels.

Interleukin-31 levels in CTCL patients were significantly higher than those in controls (*p* = 0.012) (Table 3 and Figure 1(a)). Also, SS patients had significantly higher sIL-31 when compared to patients with MF (*P* = 0.004) and to controls (*p* < 0.001). In contrast, sIL-31 did not differ significantly between MF patients and controls (*p* > 0.05) (Table 3 and Figure 1(a)).

**Figure 2:**
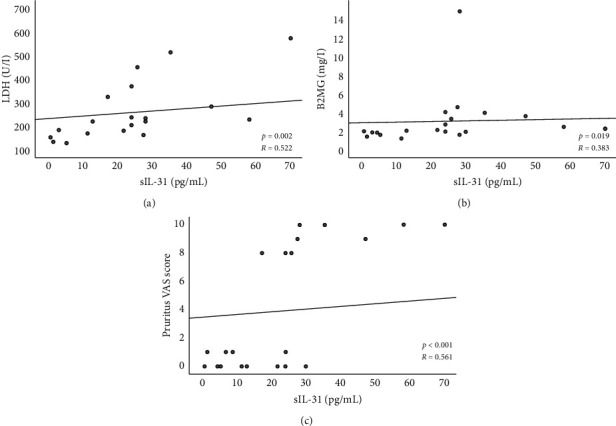
Correlations between IL-31 serum levels and LDH serum levels (a), B2MG serum levels (b), and itch VAS score (c). In this figure, 2 outliers were excluded for correlations involving sIL-31 (sIL-31 > 200 pg/mL), and the *p* and R values presented refer to analysis excluding outliers. Statistical analysis was performed using Kendall's tau-B correlation coefficient. Statistics (including/excluding the outliers): sIL-31 *vs*. LDH: *p* = 0.001; *R* = 0.545/ *p* = 0.002; *R* = 0.522; sIL-31 *vs*. B2M: *p* = 0.009; *R* = 0.406/ *p* = 0.019; *R* = 0.383; sIL-31 *vs*. SC count: *p* = 0.008; *R* = 0.406/ *p* = 0.012; *R*  =  0.423; and sIL-31 *vs*. pruritus VAS score: *p* < 0.001; *R* = 0.530/ *p* < 0.001; *R* = 0.561. B2M, beta 2 microglobulin; CTCL, cutaneous T-cell lymphoma; IL, interleukin; LDH, lactate dehydrogenase; SC, Sézary cells; and sIL-31, interleukin-31 serum levels.

Concerning CTCL stages, patients with advanced disease had significantly higher sIL-31 as compared to patients with early disease (*p* = 0.026) and to controls (*p* < 0.001) (Table 3). No significant differences were observed between patients with early disease and controls (*p* > 0.05). Moreover, sIL-31 correlated significantly with LDH (*p* = 0.001) and B2MG (*p* = 0.009) (Figures 2(a) and 2(b)).

Considering the hematological and immunophenotypic variables, CTCL patients with percentages of SC >5% of total lymphocytes had significantly higher sIL-31 levels compared to those having ≤5% (*p* = 0.008) (Table 3).

A positive correlation between sIL-31 and the number of abnormal CD4+ T cells in PB was observed, when considering all CTCL patients (*p* = 0.008). However, when analyzing only SS patients, the significance was lost.

sIL-31 was significantly higher in CTCL patients complaining of itch, as compared to those without itch (*p* = 0.021) and to controls (*p* = 0.002) (Table 3) (Figure 1(a)). Similarly, sIL-31 was significantly higher in CTCL patients reporting intense/severe itch when compared to patients reporting mild/moderate itch (*p* = 0.037) (Table 3). Correspondingly, there was a significant higher sIL-31 in CTCL patients reporting intense/severe itch, but not in CTCL patients mentioning mild/moderate itch, as compared with controls (*p* < 0.001 and *p* > 0.05, respectively) (Table 3).

In CTCL patients, sIL-31 correlated significantly with the itch VAS score (*p* < 0.001) (Figure 2(c)). When analyzing only MF patients, a relationship between sIL-31 and itch was also noted, as MF patients with intense/severe itch had significantly higher sIL-31, as compared to those with mild/moderate itch (*p* = 0.036).

### 3.2. Interleukin-8

Interleukin-8 levels did not differ significantly neither between patients and controls nor between the groups of CTCL patients mentioned above, when compared to each other and when compared to controls (*p* > 0.05 in all situations) (Table 3 and Figure 1(b)).

**Figure 3:**
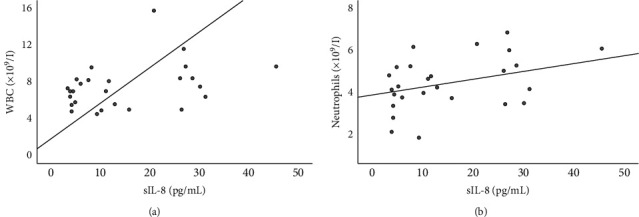
Correlations between IL-8 serum levels and peripheral blood leukocyte (a) and neutrophil (b) counts, in patients with CTCL. In this figure, 1 outlier was excluded for correlations involving sIL-8 (sIL-8 >200 pg/mL, corresponding to a patient with hyperleukocytosis). Statistical analysis was performed using Kendall's tau-B correlation coefficient. Statistics (including/excluding the outliers): sIL-8 *vs.* leukocyte count: *p* = 0.030; *R* = 0.300/ *p* = 0.085; *R* = 0.243; sIL-8 *vs*. neutrophil count: *p* = 0.021; *R* = 0.317/ *p* = 0.061; *R* = 0.262. CTCL, cutaneous T-cell lymphoma; IL, interleukin; sIL-8, interleukin-8 serum levels; and WBC, white blood cell.

Considering the hematological and immunophenotypic variables, there was a positive correlation between sIL-8 and WBC (*p* = 0.030) and neutrophil (*p* = 0.021) counts; however, after excluding one IL-8 outlier, the statistical significance was lost (Figures 3(a) and 3(b)).

No significant differences were observed in sIL-8 between CTCL patients with or without itch (*p* > 0.05), nor between these groups and controls (*p* > 0.05). However, there was a tendency to higher sIL-8 in patients classifying itch as intense/severe, compared to mild/moderate (*p* = 0.056).

## 4. Discussion

IL-31 has been associated to pruritic diseases [30–53], being consensual that it has a role in nonhistaminergic itch [22]. Our study suggests a relationship between IL-31 and CTCL disease severity and associated itch, supporting and complementing other studies [47, 49].

With respect to disease severity, we found sIL-31 to be higher in advanced CTCL cases and to correlate significantly with LDH and B2MG, which reflect tumor burden [75]. In this aspect, our results are similar to those obtained by Ohmatsu et al., who observed a positive association between sIL-31 and disease gravity in CTCL but did not investigate the relationship between sIL-31 and itch [47]. In addition, we noticed a significant positive correlation between sIL-31 and the number of PB lymphoma cells, strengthening the hypothesis that IL-31 is produced by the malignant T cells [49, 50].

Concerning itch, we found sIL-31 to be significantly higher in CTCL patients suffering from itch as compared to those without itch, just as they were significantly higher in patients with intense/severe vs. those with mild/moderate itch; we also observed a positive correlation between the sIL-31 and the itch VAS score. Our results are coherent with those obtained by Singer et al. [49], but not with those obtained in other studies [51, 52]. Indeed, Singer et al. observed that sIL-31 was higher in itching as compared to nonitching CTCL patients [49], whereas Malek et al. found that sIL-31 was higher in CTCL patients than in controls, but they did not observe significant differences between itching and nonitching cases, nor a positive correlation between sIL-31 and the itch score [51].

Previous studies have shown a Th2-biased immune response in advanced CTCL, whereas in early disease, a Th1 profile predominates [76, 77], and that leukemic CTCL cells produce mainly Th2 cytokines [78]. Taking into consideration that IL-31 is produced mostly by Th2 cells [23] and depends on IL-4 [79], the apparently discrepant results between studies can be explained by differences in patients´ characteristics. In fact, 97% of the patients studied by Singer et al. had advanced disease [49], in comparison to only 15% of the patients in Malek' study [51] and 31% in our study. In addition, the proportion of SS patients was much higher in Singer' study (70%), compared to our study (33%) and to Malek's study (3%). Möbs et al. did not observe significant differences in sIL-31 between itching and nonitching CTCL patients, despite 88% of the cases having advanced disease and 54% being SS, neither in between MF and SS patients, nor in between CTCL stages [52]. However, as stated by the authors, “only few samples exceeded the threshold allowing unequivocal sIL-31 quantification,” suggesting technical problems [52].

It is still unclear what drives IL-31 production in CTCL and the relative contribution of neoplastic and normal Th2 cells. Evidence supporting the synthesis of IL-31 by neoplastic CTCL cells was provided by three studies. Singer et al. tested CTCL patients and healthy controls for intracellular IL-31, and they found that, upon stimulation with phytohemagglutinin and ionomycin, CD4+ T cells (predominantly the neoplastic) from some CTCL patients, all of whom were pruritic, expressed intracellular IL-31 [49]. Möbs et al. observed that IL-31 mRNA was not detectable in blood tumor cells of SS patients, although SC, as normal T cells, were able to secrete IL-31 upon stimulation [52]. Finally, Cedeno-Laurent et al. found that chemokine receptor type-4-bearing T cells are a main source of IL-31 in CTCL [50]. Thus, it seems that, once activated, both normal and neoplastic T cells can produce IL-31. It could be hypothesized that the stimuli involved in T-cell activation may be infections and bacterial toxins.

Cutaneous colonization with staphylococci is common in CTCL patients and influences disease activity [55, 56], and eradication of staphylococci from the skin is associated with clinical improvement [56]. Moreover, staphylococcal superantigens were shown to induce IL-31 expression in the skin from atopic individuals, and in vitro, staphylococcal enterotoxin B induces IL-31 production by leukocytes [30].

Unlike previously observed for AD [60], in our study, sIL-8 was not significantly higher in CTCL patients than in controls, and there was no significant relation between sIL-8 and itch. Interleukin-8 is chemotactic for neutrophils [59], so, as we found, a positive correlation between sIL-8 and neutrophil (and WBC) counts would be expected.

Neutrophils are a primary line of defense against bacteria. Neutrophil serine proteases, which are released upon neutrophil activation, are major constituents of neutrophil granules [80] and key mediators of inflammation [81–83], participating in microbial destruction and influencing the immune response [80]. For instance, neutrophil-derived proteases have been shown to stimulate proinflammatory cytokines and to activate receptors implicated in itch [84–87]. Some effects of proteases in the skin have been attributed to the activation of the G-protein-coupled PAR, and the role of PAR-2 in skin inflammation and itch is well established [88–93]. Various endogenous [89–92] and exogenous [94–96] proteases, including *Staphylococcus* toxins [96], have been involved in itch. Therefore, it can be hypothesized that IL-8 may play a part in exacerbating itch in patients with CTCL by recruiting neutrophils into the tissues and promoting the release of neutrophil proteases.

## 5. Conclusions

Our study demonstrates a relationship between sIL-31 and CTCL severity and associated itch, which is frequently recalcitrant and refractory to treatment in such patients. Thus, it would make sense to develop new therapies having IL-31 and its receptor as targets, analogous to what is being carried out for AD [97]. A possible role for IL-8 in exacerbating itch in CTCL patients with concomitant infections needs to be further explored, with emphasis on the bacterial and neutrophil proteases that might be able to induce itch by acting on PAR.

## Figures and Tables

**Table 1:** Sociodemographic and clinical characteristics of the CTCL study population.

Age (years)	66 (36–90)
Gender (male/female)	14 (51.9%)/13 (48.1%)
CTCL classification	
Mycosis Fungoides	17 (63.0%)
Sézary syndrome	10 (37.0%)

Time of evaluation	
At the diagnosis	5 (18.5%)
During disease follow-up	22 (81.5%)

Skin lesions, at the time of the study	
Patches	17 (63.0%)
Plaques	8 (29.6%)
Papules	2 (7.4%)
Nodules	1 (3.7%)
Tumors	1 (3.7%)
Erythroderma‡	9 (33.3%)

Body surface area affected, at the time of the study	
<10	8 (29.6%)
[10–80%]	8 (29.6%)
≥80%	11 (40.7%)

Disease stage, at the time of the study	
Stages I and II	18 (66.7%)
Stages III and IV	9 (33.3%)

Pruritus, at the time of the study	
Yes (score 1 to 10)	18 (66.7%)
Mild/moderate (score 1 to 5)	7 (25.9%)
Intense/severe (score 6 to 10)	11 (40.7%)
VAS score, all patients	1 (0–10); 4 ± 4
VAS score, early disease/advanced disease	1 (0–10); 2 ± 3/10 (5–10); 9 ± 2
VAS score, MF patients/SS patients	0 (0–10); 2 ± 3/9 (1–10); 8 ± 3

Treatment, at the time of the study	
Directed to the disease	18 (66.7%)
Directed to itch	19 (70.4%)

Results are presented as median (range), mean ± standard deviation, and as absolute and relative frequencies. CTCL, cutaneous T-cell lymphoma; MF, Mycosis Fungoides; SS, Sézary syndrome; VAS, visual analogue scale. ‡One SS patient bore no erythroderma at the time of the evaluation.

**Table 2:** Laboratorial features of the CTCL study population, at the time of the study.

Blood cell counts and cytomorphology	
Leucocytes (x109/L)	7.30 (4.50–130.70)
Neutrophils (x109/L)	4.28 (1.84–15.82)
Lymphocytes (x109/L)	2.34 (0.20–96.20)
Monocytes (x109/L)	0.57 (0.08–17.25)
Hemoglobin (g/dL)	13.7 (8.6–16.5)
Platelets (x109/L)	220 (42–357)
Atypical lymphocytes/SC (% leucocytes)	0.0 (0.0–66.5)

Abnormal blood cell counts	
Leukocytosis (>11 × 10^9^/L)	3/27 (11.1%)
Neutrophilia (>7.0 × 10^9^/L); neutropenia (<1.5 × 10^9^/L)	1/27 (3.7%); 0/27 (0.0%)
Lymphocytosis (>3.5 × 10^9^/L); lymphopenia (<1.5 × 10^9^/L)	3/27 (11.1%); 9/27 (33.3%)
Anemia (hg < 12.0 g/dL)	7/27 (25.9%)
Thrombocytopenia (<100 × 10^9^/L)	1/27 (3.7%)

Lymphocyte immunophenotyping	
CD4+ T-cell count/*μ*L	930 (23–93329)
CD4/CD8 ratio	2.8 (0.6–99.0)
Phenotypically abnormal CD4+ SC	
% CD4+ T cells	0.0 (0.0–98.0)
% leucocytes	0.0 (0.0–70.7)
Cell counts/*μ*l	0 (0–92395)

Blood involvement	
Yes	11 (40.7%)
Stage B0 (SC < 5% lymphocytes)	17 (62.9%) †
Stage B1 (SC > 5% lymphocytes, <1000 CS/*μ*L)	5 (18.5%)
Stage B2 (SC > 1000 CS/*μ*L)	5 (18.5%)

Biochemistry	
B2MG (mg/L)	2.10 (1.20–14.70)
Increased B2MG (>2.53 mg/L)	8/23 (34.8%)
LDH (U/L)	224 (128–656)
Increased LDH (>225 U/L)	11/22 (50.0%)
Abnormal liver tests	2/27 (67.4%)
Abnormal kidney tests	1/27 (3.7%)

Results are presented as median (range) and as absolute and relative frequencies. CTCL, cutaneous T-cell lymphoma; CS, Sézary cells; B2MG, beta 2 microglobulin; LDH, lactate dehydrogenase; MF, Mycosis Fungoides; SS, Sézary syndrome. †One patient had circulating CD4+ SC, although they represent only 2.9% of CD4+ T cells, 1.8% of lymphocytes, and 0.4% of leukocytes.

**Table 3:** IL-31 and IL-8 serum levels in patients with CTCL and healthy controls.

	sIL-31 (pg/ml)	P (CTCL *vs.* C)	P (CTCL) †	sIL-8 (pg/ml)	P (CTCL *vs*. C)	P (CTCL) †
Controls (*n* = 29)	7.8 (0.3–55.3)	NA	NA	9.3 (3.4–52.0)	NA	NA
CTCL (*n* = 27)	24.2 (0.6–253.6)	**0.012**	NA	11.1 (3.4–322.5)	0.863	NA
MF (*n* = 17)	11.4 (0.6–58.6)	0.333	**0.004**	11.1 (3.4–31.2)	1.000	0.639
SS (*n* = 10)	28.3 (17.3–253.6)	**<0.001**		13.0 (4.2–322.5)	0.740	
SC ≤ 5% lymphocytes (*n* = 17)	11.4 (0.6–253.6)	0.322	**0.008**	10.1 (3.4–31.2)	0.802	0.334
SC >5% lymphocytes (*n* = 10)	28.3 (17.3–209.1)	**<0.001**		18.3 (4.2–322.5)	0.495	
SC < 1000/*μ*l (*n* = 22)	21.9 (0.6–253.6)	0.070	0.138	10.7 (3.4–31.2)	0.581	**0.033**
SC ≥ 1000/*μ*l (*n* = 5)	28.3 (17.3–60.6)	**0.003**		30.1 (6.0–322.5)	0.056	
Stages I + II (*n* = 18)	12.2 (0.6–253.6)	0.212	**0.026**	9.3 (3.4–31.2)	0.991	0.596
Stages III + IV (*n* = 9)	28.3 (17.3–209.1)	**<0.001**		15.8 (4.2–322.5)	0.736	
Without itch (VAS 0) (*n* = 9)	11.4 (0.6–30.1)	0.430	**0.021**	9.3 (3.9–31.2)	0.783	0.743
With itch (VAS 1–10) (*n* = 18)	27.7 (1.4–253.6)	**0.002**		11.4 (3.4–322.5)	0.726	
Mild/moderate itch (VAS 1–5) (*n* = 7)	7.8 (1.4–253.6)	0.749	**0.037**	8.2 (3.4–26.1)	0.253	0.056
Intense/severe itch (VAS 6–10) (*n* = 11)	28.3 (17.3–209.1)	**<0.001**		20.8 (4.2–322.5)	0.175	

Results are presented as mean ± standard deviation and as median and (range) values. Values were rounded to one decimal point. C, controls; CTCL, cutaneous T-cell lymphomas; sIL-8, interleukin-8 serum levels; sIL-31, interleukin-31 serum levels, MF, Mycosis Fungoides; NA, not applicable; PB, peripheral blood; SC, Sézary cells; SS, Sézary syndrome; VAS, visual analogue scale. †*p* values obtained when the mentioned CTCL groups were compared to each other, including outliers: MF *vs*. SS, CTCL with ≤5% SC *vs*. CTCL with >5% SC (expressed as % of PB lymphocytes); CTCL with SC < 1000/*μ*L *vs*. CTCL with SC ≥ 1000/*μ*L in the PB; CTCL stages I + II *vs*. CTCL stages III + IV; CTCL without itch (VAS 0) *vs*. CTCL with itch (VAS 1–10); CTCL with mild/moderate itch (VAS 1–5) *vs*. CTCL with intense/severe itch (VAS 6–10).

## Data Availability

The datasets used and/or analyzed during the current study are available from the corresponding author on reasonable request.

## Abbreviations

AD: Atopic dermatitis
B2MG: Beta 2 microglobulin
CTCL: Cutaneous T-cell lymphoma
EGFR: Epidermal growth factor receptor
ELISA: Enzyme-linked immunosorbent assay
EORTC: European Organization for Research and Treatment of Cancer
Hg: Hemoglobin
IL: Interleukin
ISCL: International Society for Cutaneous Lymphomas
LDH: Lactate dehydrogenase
MF: Mycosis Fungoides
PAR: Proteinase-activated receptor
PB: Peripheral blood
sIL-31: IL-31 serum levels
sIL-8: IL-8 serum levels
SC: Sézary cells
SPSS: Statistical package for social sciences
SS: Sézary syndrome
TNMB: Tumor-node-metastasis-blood
Th1: T helper 1
Th2: T helper 2
VAS: Visual analogue scale
WBC: White blood cells.
